# Race, diabetes, and cognitive function: a cross-sectional analysis of intersecting disparities in the NHANES cohort

**DOI:** 10.3389/fnins.2025.1550077

**Published:** 2025-05-06

**Authors:** Lingling Wang, Qin Zhou, Yushuang Yin, Wenqi Zhang, Chen Wang, Guanwen Lin, Duozhi Wu, Zhihua Wang

**Affiliations:** ^1^Department of Anesthesiology, Hainan General Hospital (Hainan Affiliated Hospital of Hainan Medical University), Haikou, China; ^2^Department of Anesthesiology, The First Affiliated Hospital of Sun Yat-sen University, Guangzhou, China

**Keywords:** race and ethnicity, diabetes, cognitive function, interaction, NHANES

## Abstract

**Background:**

Cognitive impairment poses a considerable challenge to public health systems worldwide, and its severity often varies depending on racial disparities. Diabetes, a prevalent chronic disease, is also known to adversely affect cognitive function. However, the interaction between race and diabetes in influencing cognitive function has not been well defined. This study aims to investigate the combined effects of race and diabetes on cognitive function using a demographically diverse group of elderly individuals.

**Methods:**

Data were drawn from the National Health and Nutrition Examination Survey (NHANES) from 2011 to 2014, involving a sample of 2,586 elderly participants aged 60 and above. Multivariate regression models were employed to assess the effects of race, diabetes status, and their interaction on cognitive test scores. Cognitive function was evaluated using the Consortium to Establish a Registry for Alzheimer’s Disease (CERAD) test, the Delayed Recall Test, the Animal Fluency Test (AFT), and the Digit Symbol Substitution Test (DSST).

**Results:**

Mexican Americans and Non-Hispanic Black people have the most frequent rates of diabetes. Non-Hispanic White people score the highest in all cognitive tests, while Mexican Americans and other Hispanics score the lowest (*p* < 0.001). Diabetic individuals score significantly lower than non-diabetics across all cognitive tests, with the most pronounced difference observed in DSST scores (*p* < 0.001). The negative association between diabetes status and DSST scores remained significant after adjusting for confounders (*p* < 0.001). Notably, the interaction between race and diabetes did not significantly influence cognitive function across the cognitive tests.

**Conclusion:**

This study found significant differences in diabetes prevalence and cognitive performance by race, along with a robust negative correlation between diabetes status and cognitive function. However, the interaction between race and diabetes does not significantly affect cognitive function.

## 1 Introduction

Cognitive impairment (CI) encompasses a spectrum of disorders, spanning the continuum from mild cognitive difficulties to advanced stages of dementia. It is characterized by a substantial deterioration in one or more areas of cognition—encompassing the realms of memorization, concentration, cognitive control, verbal skills, and perceptual abilities—sufficient to impair daily functioning without compromising consciousness (Kuipers et al., 2022). CI affects millions of older adults globally, and its prevalence is projected to increase substantially in the coming decades (GBD 2021 Diseases and Injuries Collaborators, 2024). This condition significantly impacts quality of life and places a considerable burden on both socioeconomic and healthcare systems (Hussenoeder et al., 2020; Tahami Monfared et al., 2022). By 2050, the estimated worldwide financial burden of dementia is anticipated to exceed $91.2 trillion (Jia et al., 2018). Risk factors for CI are diverse, including age, education, genetics, and chronic diseases like diabetes. Understanding the modifiable drivers of CI, such as diabetes and cardiovascular disease, is thus critical to developing targeted interventions that mitigate its global burden and promote healthy aging.

When exploring the multiple risk factors for CI, racial background is an important factor that cannot be overlooked (Mansour et al., 2020; Li et al., 2023; Wei et al., 2024). Systemic inequities amplify this risk: non-Hispanic Black people and Hispanic adults in the U.S. experience 1.5-2 times higher diabetes prevalence compared to non-Hispanic White adults (Bower et al., 2019; Cheng et al., 2019; Fang et al., 2023), coupled with 20-30% faster rates of cognitive decline (Shiekh et al., 2021). Racial disparities in diabetes management further exacerbate outcomes; for example, non-Hispanic Black individuals are 40% less likely to achieve glycemic control (HbA1c < 7%) than their White counterparts (Zakaria et al., 2023), which may accelerate neurodegeneration through prolonged metabolic dysfunction (Moran et al., 2013). These disparities are rooted in structural factors like limited healthcare access, socioeconomic marginalization, and chronic stress from systemic racism (Williams et al., 2019). Multiple studies stress that tackling such health inequities is key to achieving global health equity (Hilal and Brayne, 2022; Holt-Lunstad, 2022; Hill-Briggs and Fitzpatrick, 2023). However, current policies often neglect the combined impact of race and diabetes on cognitive health, underscoring the need for targeted research.

Diabetes itself has been demonstrated as a stand-alone predictor of cognitive impairment. Previous studies indicate that diabetes-related factors such as hyperglycemia and insulin resistance can lead to structural brain changes that increase the risk of CI (Bolo et al., 2022; Yu et al., 2023). Neuroimaging evidence reveals that diabetic patients exhibit reduced hippocampal volume and prefrontal cortex connectivity, which are key biomarkers of CI (Moran et al., 2013). These brain changes are particularly pronounced in racial minority groups (Reitz et al., 2017). Recent studies have reinforced the link between diabetes and CI, particularly affecting areas like cognitive processing velocity, executive function and recall (Cichosz et al., 2020; Casagrande et al., 2021; Jiwani et al., 2022). Additionally, pre-diabetic states have also been documented to link to CI (Makino et al., 2021; Şen et al., 2024), emphasizing the need for early diagnosis and intervention of diabetes. However, existing studies on cognitive impairment often analyze race or diabetes in isolation, and lack of nationally representative data to illustrate their synergistic effects.

Arvanitakis et al. found that while diabetes is linked to deficits in semantic memory, it spares additional cognitive areas and overall cognitive performance (Arvanitakis et al., 2010). Moreover, their study showed that the association between diabetes and cognitive ability was not different between older black and white people. However, the lack of racial diversity in this study (70% White participants) limits its ability to fully capture how race affects the interplay between diabetes and cognitive function.

To address these limitations and amplify our profound comprehension of the sophisticated interplay among race, diabetes status, and cognitive functioning, this study utilized representative NHANES data by constructing multivariate regression models incorporating a number of variables that are closely related (lifestyle factors: smoking, alcohol consumption, physical activity; socioeconomic factors: poverty-income ratio, education; and physical condition factors: age, sex, BMI, history of hypertension, history of cerebrovascular disease) were analyzed. Our analysis aims to uncover how systemic inequities and metabolic dysfunction contribute to cognitive disparities, in line with the National Institute on Aging’s priority (Hill et al., 2015). Through this comprehensive approach, we seek to provide actionable insights for developing equitable interventions to mitigate CI in high-risk populations.

## 2 Methods

### 2.1 Data sources and study participants

The study’s data originated from the NHANES, a publicly accessible national survey conducted by the National Center for Health Statistics (NCHS) of the Centers for Disease Control and Prevention (CDC). The NHANES utilizes a stratified, multistage probabilistic sampling approach to guarantee that the sampled population is reflective of the entire U.S. civilian populace, excluding those living in institutions. The survey is conducted annually and collects demographic details like years of age, gender, financial status of the household, and educational attainment, along with lifestyle factors related to health such as patterns of alcohol consumption, tobacco use, and exercise regimens, obtained via health consultations. The ethical review panel of NCHS provided clearance for the NHANES research plans, and every participant provided written affirmation of consent following disclosure. The survey’s information can be freely obtained by the public through the NHANES online portal, where one can find comprehensive information about the study’s framework, methodologies, target demographic, and available data.^1^

**FIGURE 1:**
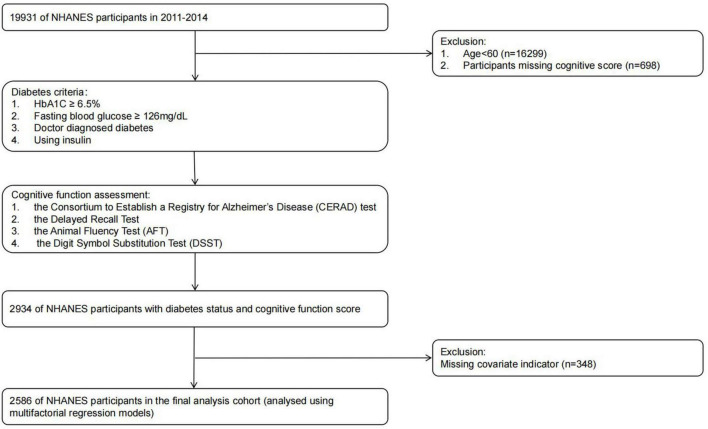
Flowchart of the participant selection from NHANES 2011–2014.

Our research analyzed NHANES data spanning the years 2011 to 2014. The NHANES data aims to provide data support for epidemiological studies and health sciences research. This Our approach ensures the representativeness and accuracy of the findings, thereby improving understanding of cognitive function in adults of different ethnic backgrounds with and without diabetes. In our analyses, we excluded subjects younger than 60 years of age, individuals lacking comprehensive cognitive data, and those with missing essential variables. The final group is made up of 2,586 adults who are aged 60 and above. Further details can be found in Figure 1.

### 2.2 Definition of variable

#### 2.2.1 Cognitive function score

Cognitive function is assessed using a variety of tests including the CERAD, the Delayed Recall Test, the AFT, and the DSST to comprehensively assess different dimensions of cognitive function. The CERAD assesses immediate learning of new linguistic information; The Delayed Recall Test assesses long-term retention of memory; the AFT assesses categorical fluency (a component of executive functioning); and the DSST assesses processing speed, sustained attention and working memory.

These tests have demonstrated good validity and reliability in previous studies. The CERAD test is highly consistent with clinical dementia diagnostic criteria and is effective in discriminating between cognitively normal and impaired individuals (Fillenbaum and Mohs, 2023). The results of the Delayed Recall Test correlate well with cognitive decline at long-term follow-up (Schneider et al., 2013). Executive dysfunction detection is marked by high sensitivity and specificity when using the AFT (Christensen et al., 2020). The DSST has excellent reliability in assessing attention and processing speed, and can accurately reflect individual differences in cognitive functioning (Zihl et al., 2014). The combination of these tests provides a comprehensive and accurate assessment of participants’ cognitive functioning.

#### 2.2.2 Diabetes

Diabetic participants were identified through criteria such as HbA1C readings of 6.5% or greater, and fasting blood glucose levels above 126 mg/dL, or affirmative responses to questions about a doctor’s diagnosis of diabetes or current insulin use.

#### 2.2.3 Smoking

Participants’ smoking habits were classified into three categories: “non-smokers,” referring to those who never consumed a minimum of 100 cigarettes; “ex-smokers,” for those exceeding 100 cigarettes in their history but currently abstaining; and “current smokers,” for those maintaining a daily habit of at least one cigarette in the last month.

#### 2.2.4 Alcohol consumption

Participants’ alcohol consumption was categorized into four categories: abstainers, those reporting 1-5 drinks per month, those with a monthly intake of 5-10 drinks, and those exceeding 10 drinks per month.

#### 2.2.5 Body mass index

Based on WHO’s standard criteria, participants were classified into four BMI ranges: below 18.5 kg/m^2^ for low weight, 18.5-24.9 kg/m^2^ for healthy weight, 25.0-29.9 kg/m^2^ for excess weight, and 30 kg/m^2^ or more for obesity.

#### 2.2.6 Cerebrovascular disease

Participants were queried with the statement: “Have you ever been informed by a medical practitioner or health expert that you suffer from congestive cardiac insufficiency, coronary artery illnesses, bouts of angina, or any form of heart disease?” The study did not include stroke survivors as they might have cognitive impairments linked to their condition.

#### 2.2.7 Hypertension

Following a 5-min period of seated rest to establish baseline blood pressure, participants underwent three successive blood pressure readings. For the purposes of this research, a systolic pressure of 130 mmHg or higher, or a diastolic pressure of 80 mmHg or higher, was classified as high. Hypertension was diagnosed if blood pressure values consistently exceeded these thresholds on two or more occasions.

#### 2.2.8 Activity levels

Physical activity was categorized into two classifications: active and inactive. Participants in the active group were identified as engaging in any exercise of moderate vigor, physical training, or sports activities, such as brisk walking, cycling, swimming, or volleyball, that leads to a minor elevation in breathing or cardiac frequency for a minimum of 10 min each week.

#### 2.2.9 Poverty income ratio

PIR is used to categorize household incomes into three different groups: Participants with low financial means (PIR ≤ 1.3), those with moderate income (1.3 < PIR ≤ 3.5), and individuals with high income (PIR > 3.5).

### 2.3 Statistical analysis

Continuous data were reported as average values (with standard deviation), whereas categorical information was depicted using counts and percentages. All statistical analyses were performed using version 4.3.1 of the R software package. Findings were classified as statistically significant whenever the *p*-value was under 0.05. The study included all patients aged 60 years or older who had full cognitive assessment outcomes, comprehensive health status details, and comprehensive information on primary variables comprising age, sex, education, alcohol consumption, tobacco use, BMI, and physical activity across both survey cycles. Among the initial cohort, 2,934 patients had complete cognitive function test data, with 2,586 having complete information on all covariates.

To investigate the correlation among race, diabetes status, and cognitive function, we developed unadjusted and adjusted regression models. The unadjusted models assessed the bivariate relationships between each independent variable (race, diabetes status) and cognitive scores. Adjusted models controlled for covariates including age, sex, education level, BMI, smoking status, drinking status, and history of cerebrovascular disease.

Interaction effects between diabetes and race on cognitive test scores were evaluated using interaction terms in the regression models. The significance of these interactions was tested at the *p* < 0.05 level.

## 3 Results

### 3.1 Headings Baseline characteristics of the research participants

Complete cognitive test data, diabetes status, and important covariates were available for 2,586 participants across the two NHANES cycles (Table 1). The majority of participants (53%) were aged 60-69 years, with a notably higher proportion of Mexican Americans in this age group (71%). Mexican Americans and Other Hispanics exhibited lower levels of education, with 39 and 25%, respectively, having less than a 9th-grade education. In contrast, 34% of Non-Hispanic White people had a college degree or higher. Among Non-Hispanic Black people, 50% were classified as obese, the highest percentage among all racial groups. Mexican Americans and Other Hispanics also exhibited a greater proportion of people possessing a PIR (Poverty Income Ratio) ≤ 1.3, accounting for 46 and 40%, respectively. The Non-Hispanic Black group had the highest percentage of current smokers (19%) compared to other groups. Additionally, 23% of Non-Hispanic Black people reported drinking alcohol more than 10 times per month, the highest proportion observed. Moreover, Non-Hispanic Black people exhibited the highest prevalence of hypertension at 64% among the ethnicities studied.

**TABLE 1:** Basic demographic characteristics of different race and ethnicity.

Characteristic	Overall (*n* = 2,586)	Mexican American (*n* = 220)	Other Hispanic (*n* = 257)	Non-Hispanic white (*n* = 1,270)	Non-Hispanic Black (*n* = 603)	Other Race (*n* = 236)	*P*-value
**Age, n (%)**							**<0.001**
60-69 years	1,308 (53%)	162 (71%)	166 (59%)	463 (51%)	386 (60%)	131 (56%)	
70-79 years	714 (27%)	36 (18%)	57 (26%)	409 (28%)	139 (27%)	73 (32%)	
80+ years	564 (20%)	22 (11%)	34 (15%)	398 (22%)	78 (14%)	32 (12%)	
**Sex, n (%)**							0.3
Female	1,334 (54%)	102 (50%)	141 (59%)	679 (54%)	296 (59%)	116 (53%)	
Male	1,252 (46%)	118 (50%)	116 (41%)	591 (46%)	307 (41%)	120 (47%)	
**Education, n (%)**							**<0.001**
Less than 9th grade	271 (5.4%)	85 (39%)	62 (25%)	56 (2.8%)	48 (7.2%)	20 (8.0%)	
9-11th grade	351 (9.9%)	26 (12%)	48 (20%)	129 (8.5%)	125 (21%)	23 (7.1%)	
High school grade/GED	602 (22%)	37 (17%)	57 (21%)	299 (21%)	175 (29%)	34 (16%)	
Some college or AA degree	754 (32%)	54 (25%)	64 (25%)	410 (33%)	169 (28%)	57 (35%)	
College graduate or above	608 (31%)	18 (7.6%)	26 (9.8%)	376 (34%)	86 (15%)	102 (34%)	
**BMI, n (%)**							**<0.001**
Underweight ( < 18.5)	36 (1.3%)	1 (0.4%)	0 (0%)	19 (1.2%)	8 (1.4%)	8 (4.1%)	
Normal (18.5 to < 25)	652 (25%)	34 (15%)	53 (19%)	316 (24%)	131 (21%)	118 (45%)	
Overweight (25 to < 30)	908 (36%)	85 (40%)	111 (44%)	465 (37%)	167 (28%)	80 (30%)	
Obese (30 or greater)	990 (38%)	100 (45%)	93 (37%)	470 (38%)	297 (50%)	30 (21%)	
**PIR, n (%)**							**<0.001**
≤ 1.3	774 (18%)	101 (46%)	104 (40%)	303 (13%)	197 (34%)	69 (26%)	
1.3-3.5	990 (38%)	79 (34%)	103 (41%)	487 (38%)	241 (41%)	80 (37%)	
> 3.5	822 (44%)	40 (19%)	50 (18%)	480 (48%)	165 (26%)	87 (37%)	
**Smoking status, n (%)**							**0.004**
Current smoker	325 (11%)	28 (12%)	28 (10%)	126 (9.7%)	123 (19%)	20 (20%)	
Former smoker	994 (40%)	82 (38%)	92 (35%)	542 (41%)	205 (32%)	73 (28%)	
Never smoker	1,267 (49%)	110 (50%)	137 (55%)	602 (49%)	275 (49%)	143 (52%)	
**Drinking status, n (%)**							**<0.001**
Non-drinker	811 (27%)	71 (33%)	89 (37%)	332 (24%)	208 (39%)	111 (41%)	
1-5 drinks/month	1,251 (48%)	113 (51%)	136 (51%)	610 (48%)	295 (46%)	97 (49%)	
5-10 drinks/month	112 (5.1%)	10 (4.0%)	5 (1.6%)	60 (5.5%)	27 (4.3%)	10 (4.6%)	
10+ drinks/month	412 (20%)	26 (12%)	27 (11%)	268 (23%)	73 (11%)	18 (5.2%)	
**History of CVD, n (%)**							0.15
No	2,203 (85%)	194 (88%)	228 (89%)	1,035 (84%)	536 (88%)	210 (88%)	
Yes	383 (15%)	26 (12%)	29 (11%)	235 (16%)	67 (12%)	26 (12%)	
**Hypertension, n (%)**							**<0.001**
No	1,152 (48%)	105 (48%)	104 (39%)	621 (50%)	221 (36%)	101 (45%)	
Yes	1,434 (52%)	115 (52%)	153 (61%)	649 (50%)	382 (64%)	135 (55%)	
**Activity levels, n (%)**							0.091
Motion	1,035 (43%)	88 (40%)	92 (35%)	507 (43%)	231 (39%)	117 (49%)	
Never	1,551 (57%)	132 (60%)	165 (65%)	763 (57%)	372 (61%)	119 (51%)	

BMI, body mass index; PIR, poverty impact ratio; CVD, cerebrovascular disease. Categorical data are represented as frequency and absolute proportion. Bold values indicate statistical significance (*p* < 0.05).

### 3.2 Prevalence of diabetes in different races

Among the 2,586 study participants, the total proportion of diabetes within the study’s population accounted for 24%. Mexican Americans and non-Hispanic Black people had higher prevalence rates of diabetes, 39 and 38%, respectively, while non-Hispanic White people had the lowest prevalence 21% (Table 2). The difference in diabetes prevalence among racial groups reached statistical significance (*p* < 0.001).

**TABLE 2:** Prevalence of diabetes in different race and ethnicity.

Characteristic	Overall (*n* = 2,586)	Mexican American (*n* = 220)	Other Hispanic (*n* = 257)	Non-Hispanic white (*n* = 1,270)	Non-Hispanic black (*n* = 603)	Other Race (*n* = 236)	*P*-value
**Diabetes status, n (%)**							**<0.001**
No	1,817 (76%)	138 (61%)	166 (65%)	970 (79%)	375 (62%)	168 (66%)	
Yes	769 (24%)	82 (39%)	91 (35%)	300 (21%)	228 (38%)	68 (34%)	

Bold values indicate statistical significance (*p* < 0.05).

### 3.3 Cognitive test scores for different races

To analyze the impact of racial diversity on cognitive performance, cognitive test scores such as CERAD test, Delayed Recall Score test, AFT test and DSST test were analyzed. As shown in Table 3, non-Hispanic white people consistently scored the highest on all tests. For the CERAD test, non-Hispanic white people had mean scores of 5.07 (Test 1), 7.08 (Test 2), and 7.87 (Test 3), totaling 20.02. In contrast, other Hispanics had the lowest scores on CERAD test, with scores of 4.24 (Test 1), 6.30 (Test 2), and 7.26 (Test 3), totaling 17.80. The delayed recall test showed a similar trend, with other races scoring the highest at 6.43 and other Hispanics scoring the lowest at 5.48. In the AFT test, non-Hispanic white people scored an average of 18.94 while other Hispanics scored an average of 15.18. In the DSST, non-Hispanic white people scored 54.78, and other Hispanics scored 37.28. Overall, non-Hispanic white people had the highest total cognitive score of 100.08, and other Hispanics had the lowest score of 75.74. These results highlight significant cognitive performance differences between races (*p* < 0.01).

**TABLE 3:** Cognitive test scores for different race and ethnicity.

Characteristic	Overall (*n* = 2,586)	Mexican American (*n* = 220)	Other Hispanic (*n* = 257)	Non-Hispanic White (*n* = 1,270)	Non-Hispanic Black (*n* = 603)	Other Race (*n* = 236)	*P*-value
CERAD: Trial 1 score	4.99 (1.68)	4.49 (1.68)	4.24 (1.60)	5.07 (1.66)	4.88 (1.74)	4.61 (1.71)	**<0.001**
CERAD: Trial 2 score	7.01 (1.76)	6.63 (1.74)	6.30 (1.87)	7.08 (1.74)	6.83 (1.76)	6.80 (1.84)	**<0.001**
CERAD: Trial 3 score	7.79 (1.68)	7.37 (1.84)	7.26 (1.78)	7.87 (1.64)	7.56 (1.74)	7.65 (1.86)	**0.002**
CERAD: Total score (3 recall trials)	19.79 (4.47)	18.49 (4.58)	17.80 (4.56)	20.02 (4.41)	19.27 (4.58)	19.06 (4.47)	**<0.001**
Delayed recall score	6.26 (2.29)	5.82 (2.35)	5.48 (2.31)	6.34 (2.29)	5.91 (2.24)	6.43 (2.09)	**0.009**
AFT score	18.28 (5.70)	16.85 (5.54)	15.18 (4.74)	18.94 (5.64)	15.00 (4.97)	16.14 (5.12)	**<0.001**
DSST score	52.40 (16.57)	40.55 (17.70)	37.28 (17.46)	54.78 (15.58)	40.62 (15.70)	51.50 (14.88)	**<0.001**
Total cognitive score	96.73 (24.24)	81.70 (24.85)	75.74 (24.32)	100.08 (23.19)	80.79 (22.37)	93.14 (21.11)	**<0.001**

CERAD, consortium to establish a registry for Alzheimer’s disease; AFT, animal fluency test; DSST, digit symbol substitution test. Continuous data are represented as means (standard deviation). Bold values indicate statistical significance (*p* < 0.05).

### 3.4 Comparison of cognitive test scores between diabetic and non-diabetic individuals of different races

As shown in Table 4, we found significant differences in median cognitive test scores between diabetic and non-diabetic patients across all racial groups by Kruskal-Wallis test, which included CERAD Total Recall Test scores, Delayed Recall Test scores, Animal Fluency Test scores, and Digit Symbol Replacement Test scores (*p* < 0.001). Further independent samples *t*-tests showed that among Non-Hispanic White people, diabetic individuals performed considerably worse than non-diabetics on all cognitive tests. In the Other Hispanic group, diabetic individuals also achieved markedly lower scores compared to non-diabetics on the Delayed Recall Test, AFT, and DDST (*p* = 0.049, *p* = 0.014, and *p* = 0.006, respectively). These findings indicate that diabetic status is significantly associated with cognitive impairment in specific ethnic groups.

**TABLE 4:** Comparison of cognitive test scores between diabetics and non-diabetics of different race and ethnicity.

Cognitive Test	Diabetes Status	Mexican American		Other Hispanic		Non-Hispanic White		Non-Hispanic Black		Other Race		*P*-value^1^
CERAD:total score (3 recall trials)	Yes	18.24 (4.08)	0.553	17.09 (4.80)	0.072	18.80 (4.33)	0.015*	18.93 (4.49)	0.675	19.26 (3.92)	0.63	**<0.001**
	No	18.60 (4.68)		18.16 (4.08)		19.51 (4.71)		19.09 (4.56)		18.96 (5.46)		
Delayed Recall Score	Yes	5.62 (2.02)	0.404	5.09 (2.43)	0.049*	5.79 (2.25)	0.012*	5.68 (2.15)	0.302	6.25 (1.92)	0.08	**<0.001**
	No	5.88 (2.44)		5.69 (2.07)		6.16 (2.37)		5.87 (2.31)		6.76 (2.26)		
AFT Score	Yes	16.02 (5.37)	0.075	14.33 (5.04)	0.014*	17.42 (5.34)	0.02*	14.82 (5.02)	0.335	15.01 (4.17)	0.614	**<0.001**
	No	17.38 (5.50)		15.91 (4.63)		18.25 (5.63)		15.22 (5.07)		15.34 (5.12)		
DSST Score	Yes	36.02 (16.91)	0.005*	33.91 (16.48)	0.006*	46.48 (14.94)	< 0.001*	38.99 (16.21)	0.059	43.76 (14.92)	< 0.001*	**<0.001**
	No	42.80 (17.41)		39.95 (17.43)		52.49 (16.18)		41.50 (14.99)		52.70 (16.33)		

CERAD, consortium to establish a registry for Alzheimer’s disease; AFT, animal fluency test; DSST, digit symbol substitution test.

*p-value (*t*-test): Represents the *p*-value based on the independent samples *t*-test, used to compare the mean cognitive scores between diabetic and non-diabetic individuals within each racial/ethnic group. A *p*-value less than 0.05 indicates that the difference in means between the two groups is statistically significant.

^1^*p*-value (Kruskal-Wallis): Represents the *p*-value based on the Kruskal-Wallis test, used to compare the median cognitive scores across different racial/ethnic groups. A *p*-value less than 0.05 indicates that at least one group’s median is significantly different from the others. If the *p*-value is less than 0.001, it is reported as “ < 0.001”. Bold values indicate statistical significance (*p* < 0.05).

### 3.5 Association of diabetic status with cognitive test scores

To further verify the impact of diabetes status on cognitive test scores, we conducted a linear regression model. Diabetic status was significantly negatively correlated with all cognitive test scores (*p* < 0.05). After controlling for variables like age group, racial background, gender identity, educational level, BMI, smoking status, drinking status, and history of cardiovascular disease (Model 2), only the negative correlation between diabetes status and DSST scores remained significant (β = –3.2, 95% CI: –5.1, –1.3, *p* < 0.006) (Table 5).

**TABLE 5:** Coefficients between diabetes status and cognitive test scores.

	Model 1			Model 2		
	**Beta**	**95% CI**	***P*-value**	**Beta**	**95% CI**	***P*-value**
CERAD: total score (3 recall trials)	–0.88	–1.5, –0.28	**0.006**	–0.16	–0.77, 0.44	0.5
Delayed recall score	–0.49	–0.78, –0.20	**0.002**	–0.25	–0.59, 0.08	0.12
AFT score	–1.5	–2.3, –0.65	**<0.001**	–0.01	–0.69, 0.68	>0.9
DSST score	–8.2	–11, –5.5	**<0.001**	–3.2	–5.1, –1.3	**<0.006**

CERAD, consortium to establish a registry for Alzheimer’s disease; AFT, animal fluency test; DSST, digit symbol substitution test; CI, confidence interval. Model1: Unadjusted. Model2: Adjusted for age, race, sex, education level, BMI, smoking status, drinking status, and history of cerebrovascular disease. Bold values indicate statistical significance (*p* < 0.05).

### 3.6 Association of racial differences with cognitive test scores

The correlation of race with cognitive test results was evident in both Model 1 (unadjusted) and Model 2 (adjusted). In the unadjusted model, the cognitive performance of non-Hispanic white people exhibited a markedly greater level compared to Mexican Americans in the reference group. The total CERAD score (β = 1.5, *p* = 0.001), AFT score (β = 2.1, *p* < 0.001) and DSST score (β = 14, *p* < 0.001) were significantly different. After adjusting for age, sex, education level, BMI, smoking status, drinking status, and history of cardiovascular disease, these associations were attenuated, especially for CERAD total score (β = 0.47, *p* = 0.3) and AFT score (β = 0.75, *p* = 0.092); however, the DSST score remained significant (β = 6.9, *p* < 0.001). In addition, non-Hispanic black people and other Hispanics also showed significant differences in cognitive test scores. In the unadjusted model, non-Hispanic Black group scored lower on the AFT (Model 1: β = –1.8, *p* < 0.001) and were closer to the reference group on the DSST (β = 0.07, *p* > 0.9). After adjustment, non-Hispanic Black people continued to score lower on the AFT (β = –2.2, *p* < 0.001) and DSST (β = –3.6, *p* = 0.006). Similarly, Other Hispanics exhibited poorer adjusted scores on the AFT (β = –1.4, *p* = 0.003) and DSST (β = –4.1, *p* = 0.003) (Table 6).

**TABLE 6:** Coefficients between race and cognitive test scores.

Race group	Model 1			Model 2		
	Beta	95% CI	*P*-value	Beta	95% CI	*P*-value
**CERAD: total score (3 recall trials)**						
Mexican American	–	–		–	–	
Other Hispanic	–0.69	–1.7, –0.33	0.2	–0.80	–1.8, 0.23	0.11
Non-Hispanic White	1.5	0.65, 2.4	**0.001**	0.47	–0.59, 1.5	0.3
Non-Hispanic Black	0.78	–0.18, 1.7	0.11	0.21	–0.72, 1.1	0.6
Other race	0.57	–0.45, 1.6	0.3	–0.37	–1.6, 0.82	0.5
**Delayed recall score**						
Mexican American	–	–		–	–	
Other Hispanic	–.33	–0.88, 0.22	0.2	–0.34	–.85, 0.17	0.2
Non-Hispanic White	0.52	0.14, 0.90	**0.009**	0.24	–0.31, 0.80	0.3
Non-Hispanic Black	0.09	–0.34, 0.52	0.7	–0.09	–0.58, 0.40	0.7
Other Race	0.62	0.05, 1.2	**0.034**	0.33	–0.48, 1.1	0.4
**AFT score**						
Mexican American	—	—		—	—	
Other Hispanic	–1.7	–2.7, –0.62	**0.003**	–1.4	–2.3, –0.64	**0.003**
Non-Hispanic White	2.1	1.1, 3.1	** < 0.001**	0.75	–0.15, 1.7	0.092
Non-Hispanic Black	–1.8	–2.8, –0.89	** < 0.001**	–2.2	–3.1, –1.4	**<0.001**
Other Race	–0.71	–2.2, 0.75	0.3	–1.8	–3.2, –0.43	**0.016**
**DSST score**						
Mexican American	—	—		—	—	
Other Hispanic	–3.3	–7.6, 1.0	0.13	–4.1	–6.3, –1.8	**0.003**
Non-Hispanic White	14	11, 17	**<0.001**	6.9	4.8, 8.9	**<0.001**
Non-Hispanic Black	0.07	–3.4, 3.5	>0.9	–3.6	–5.9, –1.3	**0.006**
Other Race	11	7.2, 17	**<0.001**	5.5	2.0, 9.0	**0.006**

CERAD, consortium to establish a registry for Alzheimer’s disease; AFT, animal fluency test; DSST, digit symbol substitution test; CI, confidence interval. Model1: Unadjusted. Model2: Adjusted for age, sex, education level, BMI, smoking status, drinking status, and history of cerebrovascular disease. Bold values indicate statistical significance (*p* < 0.05).

### 3.7 Effect of the interaction between race and diabetes on cognitive test scores

Table 7 displays the outcomes stemming from the interplay between diabetes and race on cognitive test scores. For most cognitive tests, the interaction between diabetes and race did not reach statistical significance, indicating that the impact of diabetes on cognitive performance did not demonstrate notable changes between racial groups. However, a marginally significant interaction was observed for DSST scores in the “Other race” group (*p* = 0.055).

**TABLE 7:** Effects of interaction between diabetes and race on cognitive test scores.

Cognitive test	Characteristic	Beta	95% CI	*P*-value
**CERAD: total score (3 recall trials)**	Diabetes * race			
	Diabetes * other Hispanic	–1.2	–3.3, 0.96	0.2
	Diabetes * non-Hispanic White	–0.85	–2.9, 1.2	0.3
	Diabetes * non-Hispanic Black	–0.55	–2.8, 1.7	0.5
	Diabetes * other race	–0.56	–3.4, 2.2	0.6
**Delayed recall score**	Diabetes * race			
	Diabetes * other Hispanic	–0.55	–1.9, 0.75	0.3
	Diabetes * non-Hispanic White	–0.47	–1.4, 0.49	0.2
	Diabetes * non-Hispanic Black	–0.30	–1.3, 0.73	0.4
	Diabetes * other race	–0.96	–2.3, 0.36	0.10
**AFT score**	Diabetes * race			
	Diabetes * other Hispanic	–0.77	–4.1, 2.6	0.5
	Diabetes * non-Hispanic White	–0.17	–2.3, 2.0	0.8
	Diabetes * non-Hispanic Black	0.16	–2.4, 2.7	0.9
	Diabetes * other race	1.2	–1.9, 4.2	0.3
**DSST score**	Diabetes * race			
	Diabetes * other Hispanic	–2.6	–7.8, 2.6	0.2
	Diabetes * non-Hispanic White	–3.1	–8.7, 2.4	0.2
	Diabetes * non-Hispanic Black	–0.95	–6.5, 4.6	0.6
	Diabetes * other race	–6.1	–12, 0.23	0.055

ERAD, consortium to establish a registry for Alzheimer’s disease; AFT, animal fluency test; DSST, digit symbol substitution test; CI, confidence interval.

## 4 Discussion

The findings of our study reveal significant disparities in cognitive function among various racial and ethnic groups, with Mexican Americans and Non-Hispanic Black people showing the highest prevalence of diabetes. In contrast, Non-Hispanic White group achieves the highest scores across all cognitive assessments, while Mexican Americans and other Hispanic groups consistently score the lowest. Additionally, diabetic individuals demonstrate markedly poorer performance on cognitive tests compared to their non-diabetic counterparts, with the most pronounced deficits observed in the DSST. However, the interaction between race and diabetes status does not significantly influence cognitive performance across tests. These results underscore an immediate requirement for focused actions to tackle these intersecting disparities, underscoring critical implications for public health and cognitive health outcomes in diverse populations.

Race may lead to different prevalence rates of diabetes, but there are many controversies surrounding this issue. A previous study showed that incidence rate of diabetes increased among non-Hispanic Black and Hispanic youth after the COVID-19 pandemic (Mefford et al., 2023). Another study quantified the highest diabetes prevalence at 22.1% for Hispanics and 20.4% for non-Hispanic Black people, with non-Hispanic White adults having the lowest rates at 12.1% (Cheng et al., 2019). In our study, we found that Mexican Americans and non-Hispanic Black people have the highest prevalence of diabetes, which is partially the same as the previous studies. However, a 2024 JAMA study reported the highest type 1 diabetes rates among non-Hispanic White people, followed by non-Hispanic Black people, and Hispanic people were the lowest (Fang et al., 2024). There are two possible explanations for this discrepancy. Firstly, the JAMA study focused on both children and adults with type 1 diabetes, whereas our research examined a population aged 60 and above, diagnosing diabetes without further classification. Secondly, the observed differences may be attributed to the intricate interplay of genetic susceptibilities (Ge et al., 2022) and a range of environmental determinants (Hassan et al., 2023), including socioeconomic challenges, lifestyle factors, and cultural practices, which are known to disproportionately affect ethnic minority groups and can lead to higher diabetes risk. This finding highlights the urgency of diabetes prevention and intervention measures in the elderly racial minority groups, and highlights the complexity and differences that need to be considered when studying the prevalence of diabetes in different age groups and racial classifications.

In terms of cognitive performance, various studies have consistently revealed differences in cognitive test scores between races. Existing literature indicates a lower risk for non-Hispanic White individuals to contract Alzheimer’s disease and dementia in middle age, compared to non-White adults (Weiss et al., 2023). Additionally, another study has shown that non-Hispanic White women have a lower risk of developing dementia compared to non-Hispanic Black women (Beydoun et al., 2022b). In our study, we similarly observed that Non-Hispanic White people scored higher across all cognitive tests compared to other races, which partially corroborates earlier findings. However, this disparity attenuated after adjusting for education and income (Table 6), underscoring the role of structural inequities in shaping cognitive outcomes. Factors contributing to differences in cognitive scores encompass socioeconomic status and education level, which vary significantly across different racial groups (Beydoun et al., 2022a; Rosselli et al., 2022). Furthermore, lifestyle and health behaviors, such as smoking and alcohol consumption, significantly impact cognitive abilities (Restifo et al., 2022; Van Asbroeck et al., 2024). Ethnic minorities exhibit a higher incidence of unhealthy behaviors, potentially explaining their lower cognitive scores. International studies have also reported similar disparities (Van Asbroeck et al., 2024), suggesting that these patterns are not unique to the United States but reflect broader trends related to unequal socioeconomic and health opportunities. Overall, our study highlights the importance of understanding racial differences in cognitive performance.

Diabetes consistently impairs cognitive function, leading to lower scores on cognitive tests. Previous studies have consistently indicated that diabetes is linked to poor cognitive test outcomes (Casagrande et al., 2021; Jiwani et al., 2022; Xie et al., 2024). This negative association is especially pronounced in the DSST scores of older individuals (Casagrande et al., 2021; Jiwani et al., 2022). The significant negative correlation observed between diabetes and cognitive tests in our study is consistent with existing research. Although our research findings are consistent with previous studies, we have expanded our current knowledge by studying this relationship in different racial backgrounds. The precise mechanism linking diabetes to cognitive impairment remains under investigation, but it is hypothesized that factors such as hyperglycemia-induced neuroinflammation (Zhang et al., 2022), oxidative stress (Zhang et al., 2022), insulin resistance (Kellar and Craft, 2020; Sȩdzikowska and Szablewski, 2021), and vascular issues stemming from cardiovascular disease (van Sloten et al., 2020) may play a role. These factors are known to adversely affect brain structure and function. Our study reinforces the view that diabetes constitutes a substantial contributor to cognitive decline on a worldwide scale. Therefore, it is imperative to further investigate targeted prevention strategies and interventions to mitigate cognitive impairment among individuals with diabetes.

Our analysis adjusted for key covariates including age, sex, education, BMI, poverty-income ratios, smoking, alcohol consumption, and cerebrovascular disease history. The attenuation of racial differences in CERAD and AFT scores after covariate adjustment (Table 6) suggests that these factors partially mediate the relationship between race and cognitive function. Three interrelated pathways may explain these findings: Socioeconomic Determinants: Lower education levels and higher poverty-income ratios (PIR ≤ 1.3) in Mexican Americans and Other Hispanics (Table 1) may limit access to preventive healthcare and diabetes management resources, exacerbating metabolic dysfunction and cognitive impairment. Studies show that education attainment is strongly associated with cognitive reserve, buffering against neurodegeneration (Williams et al., 2019; Beydoun et al., 2022a). Lifestyle Factors: Non-Hispanic Black participants had the highest obesity rates (50%) and smoking prevalence (19%) (Table 1). Obesity-induced insulin resistance and smoking-related vascular damage may synergistically impair cognition (van Sloten et al., 2020; Zhang et al., 2022). Biological Pathways: hypertension and cerebrovascular disease, more prevalent in racial minorities (Table 1), contribute to white matter hyperintensities and reduced cerebral perfusion, particularly affecting DSST scores that assess processing speed (Moran et al., 2013; van Sloten et al., 2020). These mechanisms align with the NIH’s emphasis on the exposome framework, which integrates biological, social, and environmental determinants of health disparities (Hill et al., 2015).

The impact of race and diabetes on cognitive function significantly influences public health, necessitating ongoing and meticulous research on their interaction. Arvanitakis et al. (2010) previously examine the interaction between races and diabetes on cognitive outcomes and found no significant link. While our analysis corroborates their results yet differs in that our study utilized a nationally representative sample with a more diverse racial spectrum, offering richer insights into how race affects the relationship between diabetes and cognition. Our findings stress the importance of adopting a more inclusive and nationally representative framework when studying the intersection of race, diabetes, and cognitive function. The lack of significant interaction between race and diabetes may reflect two competing mechanisms: (1) racial disparities in diabetes prevalence amplify cognitive risks, and (2) covariate adjustment such as socioeconomic factors attenuate these disparities. Given observed racial disparities in diabetes-related cognitive outcomes, policymakers should implement ethnoculturally tailored diabetes management programs. These programs should ensure equitable healthcare access through structural interventions and routine cognitive screening to reduce health inequities.

## 5 Strengths and limitations

The primary strength of our study lies in the utilization of NHANES, a robust, nationally representative dataset, which significantly enhances the generalizability and credibility of our findings. However, there are a number of restrictions that warrant consideration. Firstly, the lack of longitudinal data in NHANES prevents the determination of causality. Secondly, the cognitive assessments relied on specific tests that may not fully capture overall cognitive status. Additionally, the racial categorization in our sample may not encompass all ethnic groups, thereby limiting the scope of the study. Finally, potential confounders, such as dietary habits, social support, and mental health status, types and course of diabetes, were not fully accounted for, which may have impacted the evaluation of cognitive function and should be incorporated in future investigations where feasible.

## 6 Conclusion

In this study, leveraging a substantial, reflective group of senior citizens from across the United States, we identified significant differences in both diabetes prevalence and cognitive performance across racial groups. Additionally, we identified a strong negative correlation linking diabetes and cognitive function. Notably, the interaction between race and diabetes did not exert a substantial influence on cognitive outcomes. Future research should further investigate the causal relationships between these factors and develop cognitive health interventions tailored to different racial backgrounds and diabetes status. Through such nuanced and targeted research, we can better understand and address the challenges of cognitive decline, ultimately providing more equitable and effective health services to all affected populations.

## Data Availability

The raw data supporting the conclusions of this article will be made available by the authors, without undue reservation.

## Footnotes

1
https://www.cdc.gov/nchs/nhanes/index.htm

## References

[B1] Arvanitakis Z. Bennett D. Wilson R. Barnes L. (2010). Diabetes and cognitive systems in older black and white persons. *Alzheimer Dis. Assoc. Disord.* 24 37–42. 10.1097/WAD.0b013e3181a6bed5 19568148 PMC2837103

[B2] Beydoun M. Beydoun H. Banerjee S. Weiss J. Evans M. Zonderman A. (2022a). Pathways explaining racial/ethnic and socio-economic disparities in incident all-cause dementia among older US adults across income groups. *Transl. Psychiatry* 12:478. 10.1038/s41398-022-02243-y 36379922 PMC9666623

[B3] Beydoun M. Weiss J. Beydoun H. Fanelli-Kuczmarski M. Hossain S. El-Hajj Z. (2022b). Pathways explaining racial/ethnic disparities in incident all-cause and Alzheimer’s disease dementia among older US men and women. *Alzheimers Dement.* 8:e12275. 10.1002/trc2.12275 35317081 PMC8924949

[B4] Bolo N. Jacobson A. Musen G. Simonson D. (2022). Hyperglycemia and hyperinsulinemia effects on anterior cingulate cortex myoinositol-relation to brain network functional connectivity in healthy adults. *J. Neurophysiol.* 127 1426–1437. 10.1152/jn.00408.2021 35417272 PMC9109787

[B5] Bower J. Butler B. Bose-Brill S. Kue J. Wassel C. (2019). Racial/ethnic differences in diabetes screening and hyperglycemia among US women after gestational diabetes. *Prev. Chronic Dis.* 16:E145. 10.5888/pcd16.190144 31651379 PMC6824147

[B6] Casagrande S. Lee C. Stoeckel L. Menke A. Cowie C. (2021). Cognitive function among older adults with diabetes and prediabetes, NHANES 2011-2014. *Diabetes Res. Clin. Pract.* 178:108939. 10.1016/j.diabres.2021.108939 34229005 PMC8429258

[B7] Cheng Y. Kanaya A. Araneta M. Saydah S. Kahn H. Gregg E. (2019). Prevalence of diabetes by race and ethnicity in the United States, 2011-2016. *JAMA* 322 2389–2398. 10.1001/jama.2019.19365 31860047 PMC6990660

[B8] Christensen K. Gleason C. Mares J. (2020). Dietary carotenoids and cognitive function among US adults, NHANES 2011-2014. *Nutr. Neurosci.* 23 554–562. 10.1080/1028415X.2018.1533199 30326796 PMC6467741

[B9] Cichosz S. Jensen M. Hejlesen O. (2020). Cognitive impairment in elderly people with prediabetes or diabetes: A cross-sectional study of the NHANES population. *Prim Care Diabetes* 14 455–459. 10.1016/j.pcd.2019.11.005 31831376

[B10] Fang L. Sheng H. Tan Y. Zhang Q. (2023). Prevalence of diabetes in the USA from the perspective of demographic characteristics, physical indicators and living habits based on NHANES 2009-2018. *Front. Endocrinol.* 14:1088882. 10.3389/fendo.2023.1088882 36960397 PMC10028205

[B11] Fang M. Wang D. Selvin E. (2024). Prevalence of type 1 diabetes among US children and adults by age, sex, race, and ethnicity. *JAMA* 331 1411–1413. 10.1001/jama.2024.2103 38573653 PMC11040401

[B12] Fillenbaum G. Mohs R. (2023). (Consortium to establish a registry for Alzheimer’s Disease) neuropsychology assessment battery: 35 years and counting. *J. Alzheimers Dis.* 93 1–27. 10.3233/JAD-230026 36938738 PMC10175144

[B13] GBD 2021 Diseases and Injuries Collaborators (2024). Global incidence, prevalence, years lived with disability (YLDs), disability-adjusted life-years (DALYs), and healthy life expectancy (HALE) for 371 diseases and injuries in 204 countries and territories and 811 subnational locations, 1990-2021: A systematic analysis for the Global Burden of Disease Study 2021. *Lancet* 403 2133–2161. 10.1016/S0140-6736(24)00757-8 38642570 PMC11122111

[B14] Ge T. Irvin M. Patki A. Srinivasasainagendra V. Lin Y. Tiwari H. (2022). Development and validation of a trans-ancestry polygenic risk score for type 2 diabetes in diverse populations. *Genome Med.* 14:70. 10.1186/s13073-022-01074-2 35765100 PMC9241245

[B15] Hassan S. Gujral U. Quarells R. Rhodes E. Shah M. Obi J. (2023). Disparities in diabetes prevalence and management by race and ethnicity in the USA: Defining a path forward. *Lancet Diabetes Endocrinol.* 11 509–524. 10.1016/S2213-8587(23)00129-8 37356445 PMC11070656

[B16] Hilal S. Brayne C. (2022). Epidemiologic trends, social determinants, and brain health: The role of life course inequalities. *Stroke* 53 437–443. 10.1161/STROKEAHA.121.032609 35000426

[B17] Hill C. Pérez-Stable E. Anderson N. Bernard M. (2015). The national institute on aging health disparities research framework. *Ethn. Dis.* 25 245–254. 10.18865/ed.25.3.245 26675362 PMC4671408

[B18] Hill-Briggs F. Fitzpatrick S. (2023). Overview of social determinants of health in the development of diabetes. *Diabetes Care* 46 1590–1598. 10.2337/dci23-0001 37354331

[B19] Holt-Lunstad J. (2022). Social connection as a public health issue: The evidence and a systemic framework for prioritizing the “Social” in social determinants of health. *Annu. Rev. Public Health* 43 193–213. 10.1146/annurev-publhealth-052020-110732 35021021

[B20] Hussenoeder F. Conrad I. Roehr S. Fuchs A. Pentzek M. Bickel H. (2020). Mild cognitive impairment and quality of life in the oldest old: A closer look. *Qual. Life Res.* 29 1675–1683. 10.1007/s11136-020-02425-5 31993915 PMC7253517

[B21] Jia J. Wei C. Chen S. Li F. Tang Y. Qin W. (2018). The cost of Alzheimer’s disease in China and re-estimation of costs worldwide. *Alzheimers Dement.* 14 483–491. 10.1016/j.jalz.2017.12.006 29433981

[B22] Jiwani R. Dennis B. Neri A. Bess C. Espinoza S. Wang J. (2022). Type 2 diabetes independent of glycemic control is associated with cognitive impairments: Findings from NHANES. *Clin. Nurs. Res.* 31 1225–1233. 10.1177/10547738221100344 35614549 PMC10845167

[B23] Kellar D. Craft S. (2020). Brain insulin resistance in Alzheimer’s disease and related disorders: Mechanisms and therapeutic approaches. *Lancet Neurol.* 19 758–766. 10.1016/S1474-4422(20)30231-3 32730766 PMC9661919

[B24] Kuipers S. Greving J. Brunner-La Rocca H. Gottesman R. van Oostenbrugge R. Williams N. (2022). Risk evaluation of cognitive impairment in patients with heart failure: A call for action. *Int. J. Cardiol. Heart Vasc.* 43:101133. 10.1016/j.ijcha.2022.101133 36246772 PMC9563178

[B25] Li W. Zeng L. Yuan S. Shang Y. Zhuang W. Chen Z. (2023). Machine learning for the prediction of cognitive impairment in older adults. *Front. Neurosci.* 17:1158141. 10.3389/fnins.2023.1158141 37179565 PMC10172509

[B26] Makino K. Lee S. Bae S. Chiba I. Harada K. Katayama O. (2021). Diabetes and prediabetes inhibit reversion from mild cognitive impairment to normal cognition. *J. Am. Med. Dir. Assoc.* 22 1912–1918.e2. 10.1016/j.jamda.2021.02.033 33798483

[B27] Mansour O. Golden S. Yeh H. (2020). Disparities in mortality among adults with and without diabetes by sex and race. *J. Diabetes Complicat.* 34:107496. 10.1016/j.jdiacomp.2019.107496 31784284

[B28] Mefford M. Wei R. Lustigova E. Martin J. Reynolds K. (2023). Incidence of diabetes among youth before and during the COVID-19 pandemic. *JAMA Netw. Open* 6:e2334953. 10.1001/jamanetworkopen.2023.34953 37733344 PMC10514735

[B29] Moran C. Phan T. Chen J. Blizzard L. Beare R. Venn A. (2013). Brain atrophy in type 2 diabetes: Regional distribution and influence on cognition. *Diabetes Care* 36 4036–4042. 10.2337/dc13-0143 23939539 PMC3836136

[B30] Reitz C. Guzman V. Narkhede A. DeCarli C. Brickman A. Luchsinger J. (2017). Relation of dysglycemia to structural brain changes in a multiethnic elderly cohort. *J. Am. Geriatr. Soc.* 65 277–285. 10.1111/jgs.14551 27917464 PMC5311018

[B31] Restifo D. Zhao C. Kamel H. Iadecola C. Parikh N. (2022). Impact of cigarette smoking and its interaction with hypertension and diabetes on cognitive function in older Americans. *J. Alzheimers Dis.* 90 1705–1712. 10.3233/JAD-220647 36314206 PMC9988389

[B32] Rosselli M. Uribe I. Ahne E. Shihadeh L. (2022). Culture, ethnicity, and level of education in Alzheimer’s disease. *Neurotherapeutics* 19 26–54. 10.1007/s13311-022-01193-z 35347644 PMC8960082

[B33] Schneider A. Gottesman R. Mosley T. Alonso A. Knopman D. Coresh J. (2013). Cognition and incident dementia hospitalization: Results from the atherosclerosis risk in communities study. *Neuroepidemiology* 40 117–124. 10.1159/000342308 23095770 PMC3642775

[B34] Sȩdzikowska A. Szablewski L. (2021). Insulin and insulin resistance in Alzheimer’s disease. *Int. J. Mol. Sci.* 22:9987. 10.3390/ijms22189987 34576151 PMC8472298

[B35] Şen G. Tanrıkulu S. Beşer B. Akçakalem Ş Çakır S. Dinççağ N. (2024). Effects of prediabetes and type 2 diabetes on cognitive functions. *Endocrine* 85 190–195. 10.1007/s12020-024-03720-8 38358557

[B36] Shiekh S. Cadogan S. Lin L. Mathur R. Smeeth L. Warren-Gash C. (2021). Ethnic differences in dementia risk: A systematic review and meta-analysis. *J. Alzheimers Dis.* 80 337–355. 10.3233/JAD-201209 33554910 PMC8075390

[B37] Tahami Monfared A. Byrnes M. White L. Zhang Q. (2022). The humanistic and economic burden of Alzheimer’s disease. *Neurol Ther.* 11 525–551. 10.1007/s40120-022-00335-x 35192176 PMC9095804

[B38] Van Asbroeck S. Köhler S. van Boxtel M. Lipnicki D. Crawford J. Castro-Costa E. (2024). Lifestyle and incident dementia: A COSMIC individual participant data meta-analysis. *Alzheimers Dement.* 20 3972–3986. 10.1002/alz.13846 38676366 PMC11180928

[B39] van Sloten T. Sedaghat S. Carnethon M. Launer L. Stehouwer C. (2020). Cerebral microvascular complications of type 2 diabetes: Stroke, cognitive dysfunction, and depression. *Lancet Diabetes Endocrinol.* 8 325–336. 10.1016/S2213-8587(19)30405-X 32135131 PMC11044807

[B40] Wei L. Pan D. Wu S. Wang H. Wang J. Guo L. (2024). A glimpse into the future: Revealing the key factors for survival in cognitively impaired patients. *Front. Aging Neurosci.* 16:1376693. 10.3389/fnagi.2024.1376693 39026993 PMC11254678

[B41] Weiss J. Beydoun M. Beydoun H. Fanelli-Kuczmarski M. Banerjee S. Hamrah A. (2023). Pathways explaining racial/ethnic disparities in incident all-cause dementia among middle-aged US adults. *Alzheimers Dement.* 19 4299–4310. 10.1002/alz.12976 36868873 PMC10475144

[B42] Williams D. Lawrence J. Davis B. (2019). Racism and health: Evidence and needed research. *Annu. Rev. Public Health* 40 105–125. 10.1146/annurev-publhealth-040218-043750 30601726 PMC6532402

[B43] Xie Q. Nie M. Zhang F. Shao X. Wang J. Song J. (2024). An unexpected interaction between diabetes and cardiovascular diseases on cognitive function: A cross-sectional study. *J. Affect. Disord.* 354 688–693. 10.1016/j.jad.2024.03.040 38521139

[B44] Yu J. Kim R. Park S. Lee D. Cho H. Kim N. (2023). Association of long-term hyperglycaemia and insulin resistance with brain atrophy and cognitive decline: A longitudinal cohort study. *Diabetes Obes. Metab.* 25 1091–1100. 10.1111/dom.14958 36564910

[B45] Zakaria N. Tehranifar P. Laferrère B. Albrecht S. (2023). Racial and ethnic disparities in glycemic control among insured US Adults. *JAMA Netw. Open* 6:e2336307. 10.1001/jamanetworkopen.2023.36307 37796503 PMC10556965

[B46] Zhang Y. Xu W. Zhang W. Wang H. Ou Y. Qu Y. (2022). Modifiable risk factors for incident dementia and cognitive impairment: An umbrella review of evidence. *J. Affect. Disord.* 314 160–167. 10.1016/j.jad.2022.07.008 35863541

[B47] Zihl J. Fink T. Pargent F. Ziegler M. Bühner M. (2014). Cognitive reserve in young and old healthy subjects: Differences and similarities in a testing-the-limits paradigm with DSST. *PLoS One* 9:e84590. 10.1371/journal.pone.0084590 24404176 PMC3880294

